# Role of equilibrative nucleoside transporter 1 (ENT1) in the disposition of cytarabine in mice

**DOI:** 10.1002/prp2.534

**Published:** 2019-12-02

**Authors:** Jason T. Anderson, Shuiying Hu, Qiang Fu, Sharyn D. Baker, Alex Sparreboom

**Affiliations:** ^1^ Division of Pharmaceutics and Pharmacology College of Pharmacy and Comprehensive Cancer Center The Ohio State University Columbus OH USA

**Keywords:** acute myeloid leukemia, cytarabine, ENT1, NBMPR, OCTN1, transporters

## Abstract

Cytarabine (Ara‐C) is a nucleoside analog used in the treatment of acute myeloid leukemia (AML). Despite the many years of clinical use, the identity of the transporter(s) involved in the disposition of Ara‐C remains poorly studied. Previous work demonstrated that concurrent administration of Ara‐C with nitrobenzylmercaptopurine ribonucleoside (NBMPR) causes an increase in Ara‐C plasma levels, suggesting involvement of one or more nucleoside transporters. Here, we confirmed the presence of an NMBPR‐mediated interaction with Ara‐C resulting in a 2.5‐fold increased exposure. The interaction was unrelated to altered blood cell distribution, and subsequent studies indicated that the disposition of Ara‐C was unaffected in mice with a deficiency of postulated candidate transporters, including ENT1, OCTN1, OATP1B2, and MATE1. These studies indicate the involvement of an unknown NBMPR‐sensitive Ara‐C transporter that impacts the pharmacokinetic properties of this clinically important agent.

**Abbreviations**
AML: acute myeloid leukemia
Ara‐C: cytarabine
dCK: deoxycytidine kinase
LC‐MS/MS: liquid chromatography‐tandem mass spectrometry
NBMPR: nitrobenzylmercaptopurine ribonucleoside

## 1 INTRODUCTION

Acute myeloid leukemia (AML) is a form of cancer that is classified by an abnormal proliferation and differentiation of myeloid cells within the bone marrow compartment. Despite the advances in supportive care, the backbone of therapy has remained unchanged for over 30 years consisting of cytarabine (Ara‐C)‐based combination regimens.^1^ The efficacy and response to Ara‐C vary dramatically between individual AML patients and are dependent on uptake,^2^ intracellular activation,^3^ and deamination.^4^ The transport of Ara‐C is the initial step to intracellular accumulation and subsequent cytotoxicity and has been speculated to be the major contributor to clinical resistance of nucleoside analogs therapy in AML.^5^

Previous studies examining the transport of Ara‐C in AML cells indicated a correlation between Ara‐C uptake and the number of binding sites for nitrobenzylmercaptopurine ribonucleoside (NBMPR),^6^ and showed that intracellular accumulation of Ara‐C in AML cells is sensitive to inhibition by nanomolar concentrations of NBMPR.^7^ Since Ara‐C accumulation was sensitive to nanomolar concentrations of NBMPR, it was concluded that Ara‐C accumulation in leukemic cells was facilitated by an equilibrative nucleoside transporter (ENT) rather than a concentrative nucleoside transporter (CNT), which is not sensitive to nanomolar concentrations of NBMPR. Interestingly, in addition to ENT1 being sensitive to nanomolar concentrations of NBMPR, we have recently provided data for a different transporter, OCTN1, that is also sensitive to this classical nucleoside transport inhibitor.^8^ Based on these observations, it has been presumed for decades now that Ara‐C accumulates in leukemic cells by a mechanism that is dependent on the nucleoside transporter ENT1.^9^, ^10^

There have been many studies examining ENT1's role in the cellular disposition of Ara‐C, but the transport mechanism(s) contributing to the systemic disposition of Ara‐C remains poorly characterized. While no studies have directly looked at the transporter(s) contributing to systemic Ara‐C disposition, reports have shown that prior administration of NBMPR confers cytoprotection after a lethal dose of similar nucleoside analogs such as fludarabine^11^ and tubercidin,^12^ which may be attributed to decreased uptake by nucleoside transporters.

Although this thesis is consistent with the notion that concurrent administration of NBMPR causes a possible pharmacokinetic drug‐drug interaction (DDI) with Ara‐C in mice,^13^ the mechanistic details of this finding remain unclear. The aim of this current work was to reexamine the possible interaction between NBMPR and Ara‐C using mouse models that are genetically deficient for ENT1 and other putative Ara‐C carriers.

## 2 MATERIALS AND METHODS

### 2.1 Chemicals

Cytarabine, 3,4,5,6‐tetrahydrouridine, and NBMPR were purchased from Sigma‐Aldrich (St. Louis, MO). [^13^C,^15^N_2_]‐cytarabine was purchased from Alsa Chim (Illkirch Graffenstaden, France). All other chemicals were purchased from Thermo Fisher Scientific (Waltham, MA) unless otherwise specified.

### 2.2 Murine pharmacokinetic studies

All pharmacokinetic studies were conducted as previously described^14^ and with controls that were matched for age and strain: C57BL/6J for OCTN1^(−/−)^ and ENT1^(−/−)^; DBA/1lacJ for OATP1B2^(−/−)^; FVB/NJ for MATE1^(−/−)^. All mice were female, between 8 and 12 weeks of age, and were housed in a temperature‐controlled environment with a 12‐hour light/dark cycle. All mice received a standard diet and water ad libitum, and were housed and handled in accordance with the Institutional Animal Care and Use Committee of The Ohio State University and following national guidelines and regulations.

For in vivo studies, Ara‐C (dose, 15 mg/kg unless otherwise stated) was dissolved in phosphate‐buffered saline (PBS) for intraperitoneal (IP) and intravenous (IV) injection. NBMPR (dose, 100 mg/kg) was suspended in normal saline for oral gavage (PO) 1 hour prior to Ara‐C dosing; control animals received the same volume of normal saline (PO) prior to Ara‐C. At select time points after administration, blood was collected in heparinized capillary tubes from individual mice via cheek bleeding, retro‐orbital bleeding, and cardiac puncture for the final time point. Collection tubes were coated with cytidine deaminase inhibitor, 3,4,5,6‐tetrahydrouridine (THU), to prevent the metabolic degradation of Ara‐C during the collection period. Isoflurane was used as an anesthetic prior to retro‐orbital bleeds. For plasma analysis, blood samples were centrifuged at 11,000 *g* for 5 minutes, and plasma was separated and stored at –80°C until analysis by a validated method based on liquid chromatography‐tandem mass spectrometry (LC‐MS/MS).^8^ In brief, analytes of interest were extracted from plasma or whole blood using a methanol‐based method. Extracted samples were spiked with the internal standard [^13^C,^15^N_2_]‐cytarabine and then diluted with HPLC grade water. Samples were then analyzed on a Vanquish UHPLC system and a TSQ Quantum Ultra triple quadrupole mass spectrometer (Thermo Fisher Scientific). Noncompartmental pharmacokinetic parameter estimates were obtained using Phoenix WinNonlin 7.6 software (Certara, Princeton, NJ). The concentration of Ara‐C in erythrocytes was derived from the previously reported relation between whole blood and plasma concentration^15^ as follows:

$$C_{b}=H^{*}C_{\text{bc}}+{\left(1-H\right)}^{*}C_{p}.$$

In this equation, H, C_b_, C_bc_, and C_p_ represent hematocrit, blood, blood cell, and total plasma concentration of Ara‐C, respectively. Hematocrit levels were obtained from reference values reported in The Jackson Laboratory Physiological Data Summary for C57BL/6J mice.

### 2.3 Statistical analysis

An unpaired two‐tailed Student's *t* test was used to determine group differences, and *P* < .05 was considered a cutoff for statistical significance. Analyses were performed using GraphPad Prism 7.03 (La Jolla, CA).

## 3 RESULTS

### 3.1 Influence of NBMPR and ENT1‐deficiency on the disposition of Ara‐C

**Figure 1:**
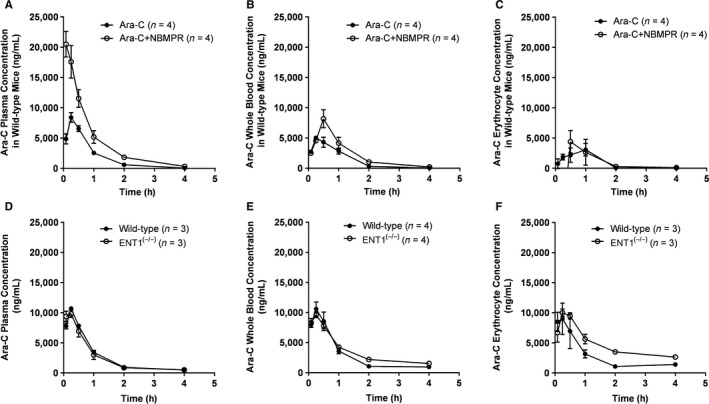
Influence of NBMPR and ENT1‐deficiency on the pharmacokinetics of Ara‐C. (A) Plasma, (B) whole blood, and (C) erythrocyte concentration‐time profiles of Ara‐C in wild‐type mice receiving vehicle (PO) (closed circles; n = 4) or 100 mg/kg NBMPR (PO) (open circles; n = 4) 1 hour prior to Ara‐C dosing. (D) Plasma (n = 3 each group), (E) whole blood (n = 4 each group), and (F) erythrocyte concentration‐time profiles of 15 mg/kg Ara‐C (IP) in wild‐type mice (closed circles; n = 4) or ENT1^(−/−)^ mice (open circles; n = 3). Results are shown as mean values (symbols) and SEM (error bars)

To better understand the transporter(s) contributing to system disposition of Ara‐C, we utilized a two‐pronged approach with pharmacologic inhibition of nucleoside transport systems using the classical nucleoside inhibitor, NBMPR, along with genetic knockout of the postulated carriers contributing to the cellular transport of Ara‐C. Similar to what was reported earlier by Cass et al, we found that NBMPR given to wild‐type mice prior to Ara‐C resulted in a 2.5‐fold (*P *= .0042) increased concentrations in plasma (Figure 1A).

Since ENT1 is highly expressed in circulating erythrocytes, we next examined the possibility that NBMPR may inhibit the distribution of Ara‐C into erythrocytes and cause altered whole blood distribution. In our murine studies, however, NBMPR only modestly increased Ara‐C concentrations in whole blood (Figure 1B) and had a negligible impact on the distribution of Ara‐C to erythrocytes (Figure 1C). Next, we wanted to evaluate the role ENT1 in the disposition of Ara‐C using our transporter‐deficient mice. After a 15 mg/kg IP dose of Ara‐C, we found that the pharmacokinetic properties of Ara‐C were not substantially altered by ENT1‐deficiency, as evidenced by the unchanged concentration‐time profiles in plasma (Figure 1D), whole blood (Figure 1E), and resulting AUCs shown in Table 1 (*P* = .61 and *P* = .12, respectively), as compared with results obtained in wild‐type mice.

**Table 1:** Pharmacokinetic parameter estimates of Ara‐C in mice

Group ID	Matrix	Dose (mg/kg)	T_1/2_ (h)	C_max_ (μg/mL)	AUC (ng × h/mL)
Wild‐type (Vehicle)	Plasma	15	0.44 (0.16)	7.79 (1.97)	7.02 (1.93)
Wild‐type (NBMPR)	Plasma	15	0.76 (0.16)	21.0 (5.32)	17.5 (4.46)
Wild‐type (Vehicle)	Whole blood	15	0.52 (0.03)	5.48 (0.901)	5.63 (1.23)
Wild‐type (NBMPR)	Whole blood	15	0.80 (0.21)	8.54 (2.39)	9.19 (2.14)
Wild‐type	Plasma	15	0.96 (0.07)	10.4 (1.01)	9.91 (2.13)
ENT1^(−/−)^	Plasma	15	0.92 (0.08)	10.7 (0.580)	10.6 (0.605)
Wild‐type	Whole blood	15	1.28 (0.31)	10.6 (2.33)	11.6 (2.41)
ENT1^(−/−)^	Whole blood	15	1.63 (0.24)	9.82 (0.826)	14.0 (0.913)
Wild‐type	Plasma	15	0.85 (0.33)	11.1 (2.13)	14.4 (4.26)
OCTN1^(−/−)^	Plasma	15	0.48 (0.10)	11.9 (2.44)	10.8 (2.03)
Wild‐type	Whole blood	15	0.52 (0.03)	5.48 (0.901)	4.32 (1.12)
OCTN1^(−/−)^	Whole blood	15	0.57 (0.06)	6.18 (1.50)	5.24 (1.16)
Wild‐type	Plasma	10	1.19 (0.11)	14.5 (2.11)	18.0 (1.17)
OATP1B2^(−/−)^	Plasma	10	1.03 (0.07)	13.4 (0.484)	14.4 (1.24)
Wild‐type	Plasma	100	0.98 (0.57)	70.8 (2.89)	106 (21.1)
MATE1^(−/−)^	Plasma	100	0.66 (0.17)	68.5 (6.70)	92.9 (13.8)

T_1/2_, half‐life of the terminal phase; C_max_, peak concentration; AUC, area under the concentration‐time curve.

Data shown as mean ± SD in parenthesis using 3‐4 animals per group.

### 3.2 Influence of OCTN1‐, OATP1b2‐, and MATE1‐deficiency on the pharmacokinetic of Ara‐C

**Figure 2:**
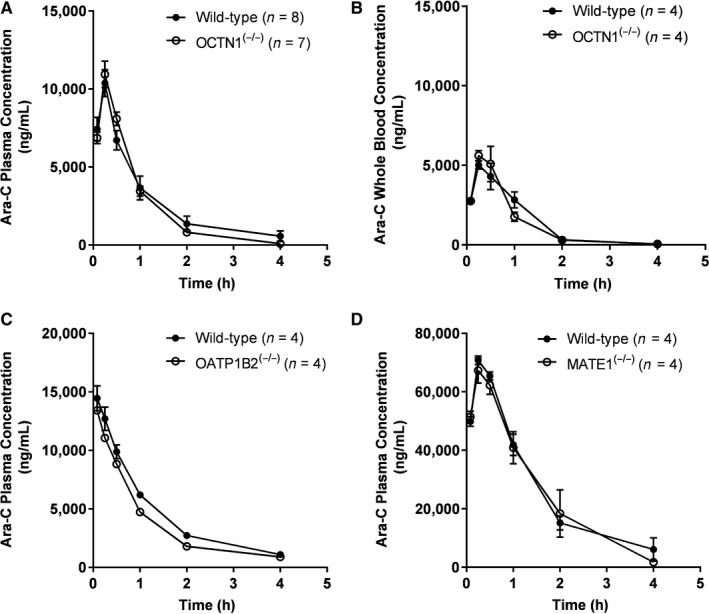
Influence of OCTN1‐, OATP1B2‐, and MATE1‐deficiency on the pharmacokinetic of Ara‐C. (A) Plasma (n = 8 for wild‐type and n = 7 for OCTN1^(−/−)^) and (B) whole blood (n = 4 each group) concentration‐time profiles of Ara‐C in wild‐type mice (closed circles) or OCTN1^(−/−)^ mice (open circles) receiving Ara‐C at a dose of 15 mg/kg (IP). (C) Plasma concentration‐time profiles of Ara‐C in wild‐type mice (closed circles; n = 4) or OATP1B2^(−/−)^ mice (open circles; n = 4) receiving Ara‐C at a dose of 10 mg/kg (IV). (D) Plasma concentration‐time profiles of Ara‐C in wild‐type mice (closed circles; n = 4) or MATE1^(−/−)^ mice (open circles; n = 4) receiving Ara‐C at a dose of 100 mg/kg (IP). Results are shown as mean values (symbols) and SEM (error bars)

To evaluate the alternative transport mechanisms involved in the NBMPR‐Ara‐C interaction, we next considered a possible contribution by the ergothioneine transporter, OCTN1. Similar to our ENT1 pharmacokinetic studies, we repeated this format in wild‐type and OCTN1^(−/−)^ mice but found that OCTN1‐deficiency did not influence the levels of Ara‐C in plasma (Figure 2A) or whole blood (Figure 2B). Despite the involvement of the liver and kidney in regulating the systemic exposure to Ara‐C, we found that the plasma levels of Ara‐C were not significantly altered by the deficiency of OATP1B2 (Figure 2C) or MATE1 (Figure 2D).

## 4 DISCUSSION

Despite the many years of clinical use, the transporter(s) impacting the systemic disposition properties of Ara‐C remains poorly understood. To gain insights into this field, we took advantage of a previously reported, possible DDI between Ara‐C and NBMPR, an agent with potent inhibitory properties toward the putative Ara‐C uptake transporter, ENT1, which we were able to recapitulate in both plasma (Figure 1A) and whole blood (Figure 1B). We opted to examine both plasma and whole blood in this study due to the notion that erythrocytes have a known ability to sequester similar antimetabolites such as 5‐fluorouracil,^16^ and that the antiviral nucleoside analog, ribavirin, accumulates into erythrocytes via an ENT1‐mediated mechanism.^17^ Interestingly, Cass et al did report similar pharmacokinetic alterations in their mouse model but dismissed these findings as insignificant due to its impact on therapeutic potentiation rather than a mechanistic basis as is the aim of this study. Of note, we also noticed a slight increase in half‐life after the addition of NBMPR, which may have arisen from a decreased uptake into a compartment of high Ara‐C metabolism, such as the liver,^18^ rather than an intrinsic ability of NBMPR to inhibit the deamination reaction of Ara‐C.

After confirming the presence of a possible DDI with the classical nucleoside inhibitor, NBMPR, and Ara‐C, we wanted to test if this increase in exposure was due to the inhibition of a nucleoside transporter. Currently, there is a lack of publication directly addressing the nucleoside transporter(s) contributing to the systemic disposition of Ara‐C using an in vivo model. In vitro data show the role of ENT1 and to a lesser extent, ENT2, to contribute to cellular uptake of Ara‐C.^9^ In regard to other nucleoside transporters contribution in our mouse model, a dose of 15 mg/kg NBMPR (IP) generated NBMPR plasma levels >1 µmol/L and was shown to accumulate in RBCs.^19^ With this in mind, NBMPR could potentially inhibit other nucleoside transporters, such as CNTs, but based upon in vitro data, ENTs contribute to cellular disposition of Ara‐C to a much greater extent than CNTs.^9^, ^10^

In our study and contrary to the literature that suggests Ara‐C is a substrate of ENT1, removal of the transporter has no impact on systemic Ara‐C disposition in both plasma (Figure 1D) and whole blood (Figure 1E). This apparent lack of erythrocytes contributing to the in vivo blood distribution of Ara‐C (Figure 1F) is consistent with the finding that Ara‐C does not substantially interact with erythrocyte membranes in a nonspecific manner,^20^ and with results from a recent study indicating that the contribution of ENT1 to the cellular uptake of Ara‐C in vitro is minimal.^8^

Since we were unable to recapitulate an increase in Ara‐C exposure as seen in the possible NBMPR DDI with ENT1 deficiency, we wanted to explore other transport systems contributing to Ara‐C disposition, such as OCTN1. In support of this notion, OCTN1 is highly expressed in erythrocytes,^21^ has been previously linked with Ara‐C transport in myeloid cells, and is sensitive to inhibition by NBMPR.^8^ Involvement of OCTN1 would also be consistent with the previous finding that opossum kidney proximal tubular cells express an (unidentified) organic cation transporter that recognizes tetraethylammonium (TEA), and is sensitive to inhibition by several nucleosides, including Ara‐C.^22^ Interestingly, we saw no difference in the systemic disposition in plasma (Figure 2A) or whole blood (Figure 2B) between OCTN1^(−/−)^ and wild‐type mice. This is somewhat unexpected given that genetic deficiency of OCTN1 was previously associated with altered plasma levels and uptake of substrates such as ergothioneine in organs of elimination (eg, kidney and liver) and distribution (eg, heart).^23^ It is possible that the inability to translate this documented in vitro uptake data in cell‐based models to an in vivo scenario is caused by a species‐dependent interaction between Ara‐C and human or mouse OCTN1. Such interspecies differences in uptake mechanisms for xenobiotics have been previously recorded for various drug‐transporter pairs, including sorafenib^24^ and digoxin.^25^

In this context, it is worth pointing out that the intracellular accumulation of Ara‐C was recently found to not be influenced in a cell‐based model engineered to overexpress human OCTN1.^26^ In this analysis, the authors used an LC‐MS/MS‐based method to measure the intracellular levels of unchanged Ara‐C, whereas in our original studies, we used radiolabeled drug and analyzed total radioactivity [ie, the total of parent drug and metabolite(s)]. This is an important methodological difference as Ara‐C can undergo rapid enzyme‐mediated metabolism once inside cells to form mono‐, di‐, and tri‐phosphorylated forms^27^ that may easily escape detection and result in underestimating the actual extent of uptake. The extensive formation of phosphorylated Ara‐C metabolites was previously demonstrated in the HEK293 cells used in our experiments.^8^ Using the same model, we have now confirmed in a comparative analysis that intracellular levels of total radioactivity originating from Ara‐C in cells overexpressing OCTN1 are high, while levels of the unchanged parent drug as measured by LC‐MS/MS remain undetectable (Figure S1).

As a next step toward understanding the mechanisms underlying the NBMPR‐Ara‐C interaction, we evaluated the contribution of the liver and kidney as important organs of elimination. OATP1B2, an uptake transporter localized to the liver, has been previously described as a transporter of Ara‐C using in vitro model systems.^28^ This supports the possibility that OATP1B2 could act as a putative carrier of relevance to drug interactions with Ara‐C. In addition, we explored a potential connection with the renal transporter MATE1, based on the previous observation that concurrent administration of the nucleoside analog, clofarabine, with either Ara‐C or fludarabine, results in a marked change in clofarabine clearance compared to clofarabine given alone, suggesting an interaction at the level of a renal apically localized transporter.^29^ A connection with MATE1 is further supported by the notion that certain antiviral nucleosides are transported by organic cation transporters,^30^ and a direct connection has been suggested for rodent organic cation transporters in relation to Ara‐C.^31^ In evaluating these alternative mechanisms, only plasma samples were obtained in the pharmacokinetic studies involving OATP1B2‐ or MATE1‐deficiency due to the minimal expression of these transporters outside of the liver^32^ and kidney,^33^ respectively. These findings support the notion that, in addition to the Ara‐C transporter ABCC4,^28^ MATE1 can now be discounted as a key renal tubular secretion/reabsorption pathway for Ara‐C that is liable to clinically relevant DDIs.

One plausible rationale for the inability to translate our in vitro findings into in vivo models could be explained by compensatory changes following the genetic deletion of a specific transporter in mice. In regard to our ENT1 model, there are no significant compensatory changes noted in gene expression of nucleoside transporter or metabolism after genetic deletion of murine ENT1.^34^, ^35^ Likewise, previous studies have indicated that genetic deficiency of OATP1B2 is not associated with any pronounced compensatory alterations in metabolic enzyme or transporter expression in the liver and kidney.^36^ Similar findings have been reported for MATE1‐knockout mice^37^ and OCTN1‐knockout mice.^38^ Taken together, compensatory changes in our murine models cannot account for the inability to detect changes in the systemic disposition in our putative Ara‐C transporter‐deficient mice.

In conclusion, this study confirms the existence of a possible NBMPR‐mediated interaction with the nucleoside analog Ara‐C in mice that appears to occur independently of two known NBMPR‐sensitive Ara‐C transporters (ENT1 and OCTN1) and is unlikely connected with two other transporters of suspected relevance (OATP1B2 and MATE1). The discrepancy between previously reported in vitro observations and those observed here in mice supports the possibility that one or more additional uptake transporters for Ara‐C exist in mice that are highly sensitive to inhibition by NBMPR. Although the identity of the carrier‐mediated mechanism(s) remains unconfirmed and requires further investigation, this study provides direct in vivo evidence that ENT1 is a transporter of limited clinical relevance to the systemic pharmacokinetics of Ara‐C.

## DISCLOSURE

The authors declare that there is no conflict of interest.

## ETHICS STATEMENT

All statements in this article were constructed with the appropriate ethical considerations. All animals were housed and handled in accordance with the Institutional Animal Care and Use Committee of The Ohio State University and following national guidelines and regulations.

## DATA REPOSITORY LINK

A data repository link is not applicable to the contents of this manuscript.
