# Supplementary material for: Role of equilibrative nucleoside transporter 1 (ENT1) in the disposition of cytarabine in mice
Source: Pharmacol Res Perspect. 2019 Dec 2;7(6):e00534. doi: 10.1002/prp2.534 (PMC6887677; doi:10.1002/prp2.534)

**Supplemental Figure Legend**

**Supp. Fig. 1:** Measurement of the unchanged parent compound, Ara-C, in HEK293 cells transfected with OCTN1 using LC-MS/MS. Chromatograms are shown for a sample of Ara-C in uptake media containing the drug at a concentration of 1 µM (closed circles) or lysates of HEK293 cells overexpressing OCTN1 (open circles) following a 30-min incubation of the cells with Ara-C (extracellular concentration, 1 µM) at 37°C.

**Suppl Figure 1**


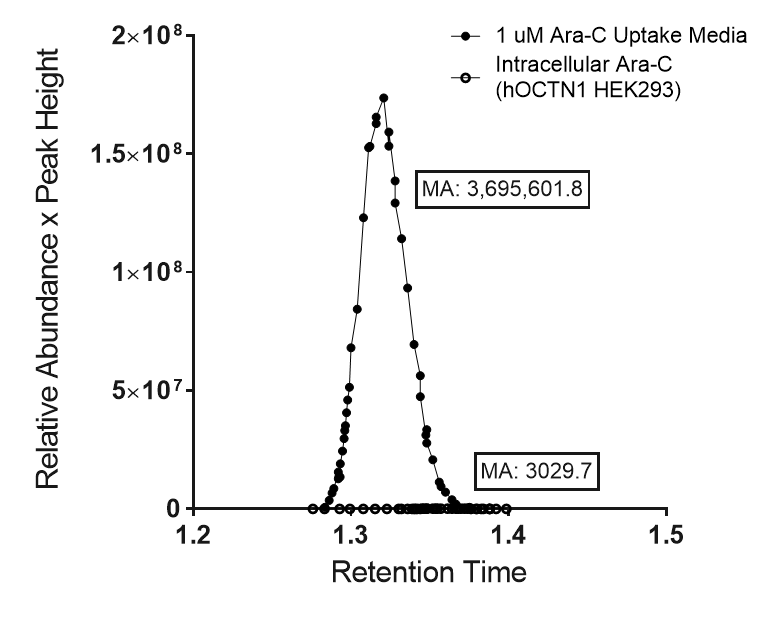

Supplement: Supplementary file 1 [file PRP2-7-e00534-s001.docx]
