# Supplementary figures and images for: Role of equilibrative nucleoside transporter 1 (ENT1) in the disposition of cytarabine in mice
Source: Pharmacol Res Perspect. 2019 Dec 2;7(6):e00534. doi: 10.1002/prp2.534 (PMC6887677; doi:10.1002/prp2.534)

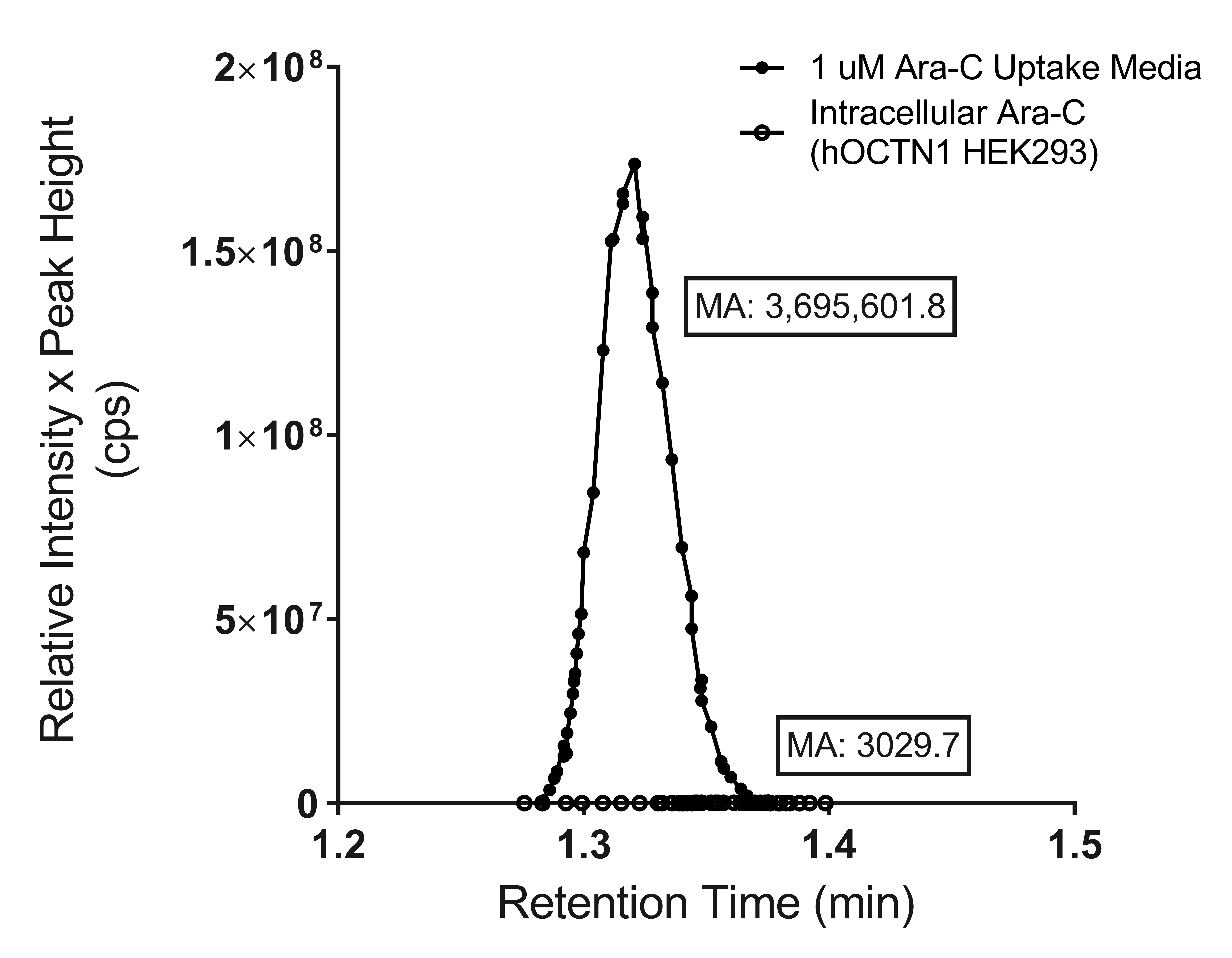

Supplement: Supplementary file 2 [file PRP2-7-e00534-s002.jpg]
